# Bilateral versus Single Internal Mammary Coronary Artery Bypass Grafting in Sweden from 1997–2008

**DOI:** 10.1371/journal.pone.0086929

**Published:** 2014-01-21

**Authors:** Magnus Dalén, Torbjörn Ivert, Martin J. Holzmann, Ulrik Sartipy

**Affiliations:** 1 Department of Cardiothoracic Surgery and Anesthesiology, Karolinska University Hospital, Stockholm, Sweden; 2 Department of Molecular Medicine and Surgery, Karolinska Institutet, Stockholm, Sweden; 3 Department of Emergency Medicine, Karolinska University Hospital, Stockholm, Sweden; 4 Department of Internal Medicine, Karolinska Institutet, Stockholm, Sweden; Istituto Clinico S. Ambrogio, Italy

## Abstract

**Background:**

Prior observational studies have suggested better outcomes in patients who receive bilateral internal mammary arteries (BIMA) during coronary artery bypass grafting (CABG) compared with patients who receive a single internal mammary artery (SIMA). The aim of this study was to analyze the association between BIMA use and long-term survival in patients who underwent primary isolated CABG.

**Methods and Results:**

Patients who underwent primary isolated non-emergent CABG in Sweden between 1997 and 2008 were identified. The SWEDEHEART registry and other national Swedish registers were used to acquire information about patient characteristics and outcomes. Unadjusted and multivariable adjusted regression models were used to estimate the association between BIMA use and early mortality, long-term survival, and a composite of death from any cause or rehospitalization for myocardial infarction, heart failure, or stroke in the overall cohort and in a propensity score-matched cohort. The study population consisted of 49702 patients who underwent CABG with at least one internal mammary artery, and 559 (1%) of those had BIMA grafting. In the adjusted analyses, BIMA use was not associated with better survival compared with SIMA use in the overall cohort (hazard ratio (HR) for death: 1.16, 95% confidence interval (CI): 0.97 to 1.37) or in the matched cohort (HR: 1.04, 95% CI: 0.78 to 1.40). The results were similar for early mortality and the composite endpoint. Reoperation for sternal wound complications was more common among BIMA patients (odds ratio: 1.71, 95% CI: 1.01 to 2.88).

**Conclusions:**

BIMA grafting was performed infrequently and was not associated with better outcomes compared with SIMA grafting in patients undergoing non-emergent primary isolated CABG in Sweden during 1997–2008.

## Introduction

Prior observational studies have suggested improved morbidity and mortality rates in patients who receive bilateral internal mammary arteries (BIMA) during coronary artery bypass surgery (CABG) compared with patients who receive a single internal mammary artery (SIMA) [1]–[13]. In the only randomized trial comparing survival after BIMA versus SIMA use, preliminary results demonstrated no difference in one-year mortality but a small increase in sternal wound complications after BIMA grafting [8]. In 2011, BIMA grafting to non-LAD coronary arteries received a class IIa recommendation (level of evidence grade B) by the American College of Cardiology Foundation/American Heart Association guidelines for CABG, to improve survival and decrease reintervention rates [9]. The 2010 European Society of Cardiology/European Association for Cardiothoracic Surgery guidelines on myocardial revascularization state that complete revascularization with arterial grafting to non-LAD coronary systems is indicated in patients with reasonable life expectancy [10]. This statement received a class I A recommendation even though no randomized trial has been conducted to support this recommendation. Although there are many data indicating a survival benefit for patients undergoing CABG with BIMA use, this surgical strategy seems underutilized. The right internal mammary artery has been named the forgotten conduit, and BIMA grafting is currently used in less than 10% of patients undergoing CABG in the United Kingdom and Ireland and 4% in the USA [3], [11], [12], [14], [15].

We performed a nationwide population-based cohort study to investigate time-related trends for BIMA use and long-term survival after BIMA-CABG in Sweden during 1997 to 2008. The primary aim was to analyze the association between BIMA use and long-term survival in patients who underwent primary isolated non-emergent CABG. A secondary objective was to study the association between BIMA-CABG and a composite end-point of rehospitalization for myocardial infarction, heart failure, stroke, or death from any cause. We also investigated early mortality and BIMA use by year and region in Sweden during the study period.

## Methods

### Design

We conducted a nationwide population-based cohort study.

### Ethics statement

The study was approved by the regional Human Research Ethics Committee, Stockholm, Sweden. Patient consent was waived because data were analyzed anonymously.

### Study population

We identified all patients who underwent CABG in Sweden between 1997 and 2008 from the SWEDEHEART (Swedish Web-system for Enhancement and Development of Evidence-based care in Heart disease Evaluated According to Recommended Therapies) registry [16]. We excluded patients who had undergone previous cardiac surgery, had concomitant procedures in addition to CABG, underwent surgery within 24 hours of decision, those in whom no internal mammary artery (IMA) was used, and patients with single grafts. The final study population consisted of patients who underwent primary isolated non-emergent CABG with at least one IMA.

### Data sources and definitions

The Swedish unique personal identity number [17] was used by the National Board of Health and Welfare to retrieve information from the national registers to assemble the study database. Baseline patient characteristics were obtained from SWEDEHEART and the Swedish National Patient Register (Swedish National Board of Health and Welfare) [18], [19]. The National Patient Register covers all diagnoses for all patients hospitalized in Sweden from 1987. All patients with a pre- or post-operative diagnosis of myocardial infarction (International Classification of Disease version 10 [ICD-10] code I21), heart failure (ICD-10 code I50), or stroke (ICD-10 codes I60 to I69) were identified. The validity of these diagnoses in the Swedish National Patient Register has been evaluated and was found to be 95% for a primary diagnosis of heart failure; the positive predictive value was 98.6% for stroke and 98%–100% for myocardial infarction [18], [19]. Diabetes mellitus was defined as ongoing treatment with insulin or oral hypoglycemic medication. Peripheral vascular disease was defined as a history of claudication, carotid artery stenosis >50%, or previous or planned vascular surgery. Chronic obstructive pulmonary disease was defined as daily use of steroids or bronchodilators. Left ventricular function was categorized as severely reduced (ejection fraction <30%), reduced (ejection fraction 30–50%), or normal (ejection fraction >50%). The additive European System for Cardiac Operative Risk Evaluation (EuroSCORE) was calculated as previously described and was included in the SWEDEHEART register from 2001 [20]. Glomerular filtration rates (eGFR) were estimated using the simplified Modification of Diet in Renal Disease study equation [21].

### Outcome measures

The primary outcome measure was all-cause mortality. Secondary outcome measures included early mortality, defined as death within 30 days from surgery, and a composite endpoint of rehospitalization for myocardial infarction, heart failure, stroke, or death from any cause. Survival status was ascertained in February 2011 using the Swedish personal identity number [17] and the continuously updated Total Population Register at Statistics Sweden. Follow-up regarding myocardial infarction, heart failure, and stroke ended on December 31, 2008.

### Statistical analysis

The study database was created by the Swedish National Board of Health and Welfare by linking information in the previously mentioned national registers using the Swedish personal identity number. Time-to-event was calculated as the time in days from the date of surgery until death from any cause for the primary end-point, and for the secondary composite end-point, we calculated the time in days from surgery until rehospitalization for myocardial infarction, heart failure, stroke, or death from any cause, whichever came first. We used the Kaplan-Meier method to calculate cumulative survival and construct survival curves and the log-rank test to compare differences between the curves.

The SIMA to BIMA hazard ratio (HR) was estimated by Cox proportional hazards regression models, first without adjustment and second with adjustment for age and sex. We then performed additional adjustments for other baseline characteristics and considered all variables listed in Table 1 and postoperative events. We used a manual forward and backward stepwise selection strategy for model selection. The final multivariable model included the following variables: age (continuous), gender (male/female), eGFR (four categories: 1 [reference category]: >60, 2: 45 to 60, 3: 30 to 45, and 4: 15 to 30 mL/min/1.73 m^2^), left ventricular ejection fraction (three categories: 1 [reference category]: normal [ejection fraction >50%], 2: reduced [ejection fraction 30 to 50%], and 3: severely reduced [ejection fraction <30%]), diabetes mellitus (no/yes), chronic obstructive pulmonary disease (no/yes), peripheral vascular disease (no/yes), preoperative myocardial infarction (no/yes), preoperative stroke (no/yes), preoperative heart failure(no/yes), perioperative acute kidney injury (no/yes), and use of cardiopulmonary bypass during surgery (yes/no).

**Table 1:** Baseline characteristics of the overall cohort.

	All patients	SIMA	BIMA
Number of patients	49702	49143	559
Percent of study population	100	98.9	1.1
Age, mean (SD), years	66.7 (9.2)	66.7 (9.2)	64.4 (11.1)
Female sex (%)	21.0	21.0	25.9
Estimated GFR, mean (SD), mL/min/1.73 m^2^	75 (21)	75 (21)	75 (22)
Diabetes mellitus (%)	22.7	22.8	13.7
Hypertension (%)	56.1	56.1	51.3
Hyperlipidemia (%)	57.6	58.5	57.6
Peripheral vascular disease (%)	7.3	7.3	13.6
Current smoking (%)	18.9	19.1	18.9
Chronic obstructive pulmonary disease (%)	4.6	4.6	4.3
Prior myocardial infarction (%)	42.9	42.9	42.8
Prior stroke (%)	4.3	4.3	3.9
Heart failure (%)	3.7	3.7	2.7
Left ventricular function			
Ejection fraction >50% (%)	73.5	73.6	70.4
Ejection fraction 30–50% (%)	23.3	23.3	25.3
Ejection fraction <30% (%)	3.2	3.2	4.3
Surgery within 7 days of decision (%)	28.2	28.2	32.4
CABG without cardiopulmonary bypass (%)	6.3	6.2	18.6
No. of grafted coronary arteries, mean (SD)	3.5 (0.95)	3.5 (0.95)	3.1 (0.90)
Radial artery used (%)	5.7	5.5	16.2
Acute perioperative kidney injury (%)	13.2	13.2	9.2

BIMA = bilateral internal mammary artery, GFR = glomerular filtration rate, CABG = coronary artery bypass grafting, SD = standard deviation, SIMA = single internal mammary artery. Acute perioperative kidney injury was defined as a >0.3 mg/dL (26 µmol/L) increase in postoperative creatinine values.

Unconditional logistic regression models were used to calculate odds ratios (OR) and 95% CIs for the association between BIMA use and early mortality. We performed an unadjusted analysis and then adjusted for EuroSCORE; finally, a full multivariable model was created.

Some data were missing for the following baseline variables: preoperative left ventricular function (30%), diabetes mellitus (35%), peripheral vascular disease (30%), eGFR (16%), and acute perioperative kidney injury (35%). Multiple imputation by chained equations was used to impute missing values. The event indicator and the Nelson-Aalen estimator of the cumulative baseline hazard were included in the imputation model [22]. One hundred datasets were imputed, and estimates from these datasets were combined.

To reduce selection bias, we calculated propensity scores for each patient in the imputed dataset by logistic regression, with BIMA use as the dependent variable. In the overall cohort, we used the propensity score for regression adjustment by introducing the propensity score as a continuous covariate in the final multivariable model and also for stratification by dividing the propensity score into quintiles and then stratifying based on the propensity score quintile.

We then constructed a propensity score matched cohort by nearest neighbor matching without replacement, one SIMA patient to one BIMA patient, in which the propensity score differed by no more than 0.001. We estimated standardized differences for variables after matching to investigate post-match balance. Standardized differences <10% are generally considered a small and acceptable imbalance. In the matched cohort, we used conditional logistic regression to assess the association between BIMA use and early mortality and Cox proportional hazards regression stratified based on matched pairs for the estimation of HRs for long-term survival and the composite end-point of rehospitalization or death.

Stata version 12.1 (StataCorp LP, College Station, TX, USA) was used for all analyses.

## Results

### Study population and baseline characteristics

From the SWEDEHEART register, we identified 69241 adult patients who underwent CABG between January 1997 and December 2008. We excluded 1234 patients who had previous cardiac surgery, 9509 patients who had another cardiac procedure in addition to CABG, 2434 patients who underwent emergency surgery, defined as surgery within 24 hours of decision, and 6362 patients in whom an internal mammary artery was not used or who had less than two grafted coronary arteries. The final study population consisted of 49702 patients who underwent primary isolated non-emergent CABG with at least one IMA. In total, 559 patients had undergone CABG with BIMA grafting and 49143 with SIMA grafting. The baseline characteristics of the study population are shown in Table 1. The BIMA and SIMA groups were not balanced regarding the potentially confounding factors of diabetes mellitus, peripheral vascular disease, and the use of off-pump coronary artery bypass surgery.

### Follow-up and early outcomes

The total follow-up time was 370596 patient-years (mean 7.5 years). During follow-up, 137 (25%) patients in the BIMA group died compared with 11798 (24%) patients in the SIMA group. Early mortality, defined as death within 30 days of surgery, was 1.8% (10/559) in the BIMA group and 1.4% (680/49143) in the SIMA group. There was no significant association between BIMA use and early mortality; the unadjusted OR was 1.30 (95% CI: 0.69 to 2.44), the EuroSCORE adjusted OR was 1.77 (95% CI: 0.93 to 3.36), and the full multivariable model OR was 1.29 (95% CI: 0.51 to 3.23). There was a significant association between BIMA use and reoperation for sternal wound complications (adjusted OR: 1.71, 95% CI: 1.01–2.88) but not for reoperation for bleeding (adjusted OR: 1.18, 95% CI: 0.71–1.95).

### Association between BIMA use and all-cause mortality in the overall cohort

The crude and multivariable adjusted associations between BIMA use and all-cause mortality are shown in Table 2.

**Table 2:** Crude and multivariable adjusted association between BIMA use and all-cause mortality in 49702 patients who underwent non-emergent primary isolated CABG during 1997 to 2008 in Sweden.

	SIMA*	BIMA
Number of patients	49143	559
Number of deaths (%)	11798 (24)	137 (25)
	HR (95% CI)	
Crude	1.00	0.95 (0.80–1.13)
Adjustment for age and sex	1.00	1.12 (0.94–1.32)
Multivariable model**	1.00	1.16 (0.97–1.37)

Reference category.

Multivariable adjustment was made for age, gender, estimated glomerular filtration rate, left ventricular ejection fraction, diabetes mellitus, chronic obstructive pulmonary disease, peripheral vascular disease, preoperative myocardial infarction, stroke, heart failure, perioperative acute kidney injury, and the use of cardiopulmonary bypass during surgery.

BIMA = bilateral internal mammary artery, CABG = coronary artery bypass grafting, CI = confidence interval, HR = hazards ratio, SIMA = single internal mammary artery.

#### Unadjusted analysis

**Figure 1:**
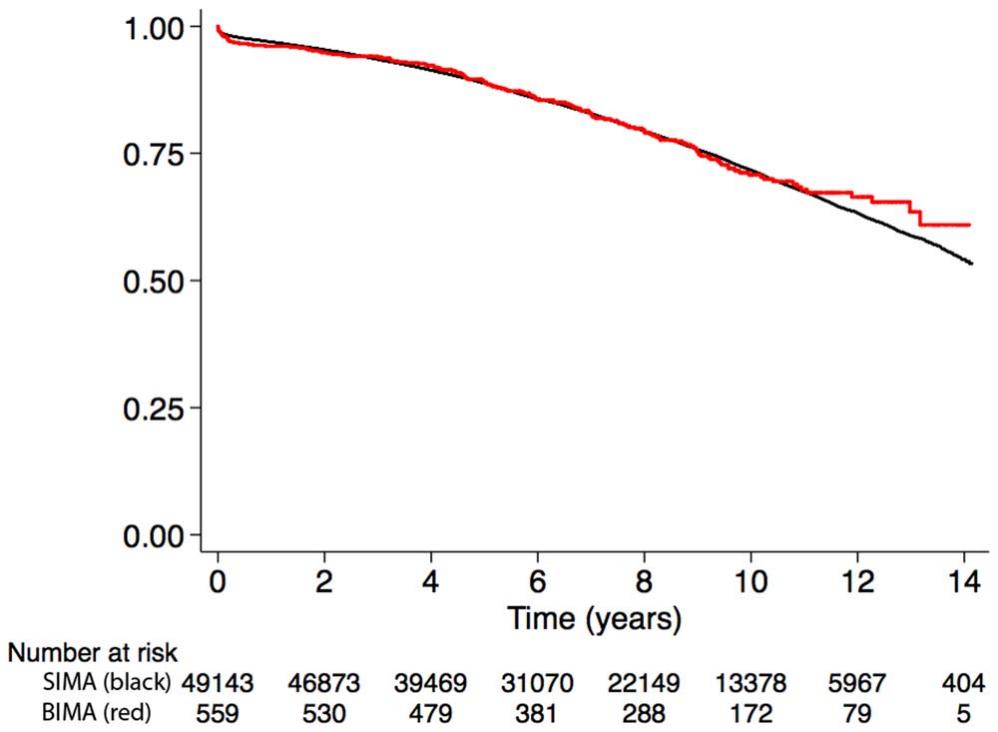
Kaplan-Meier survival in the overall cohort.

In the unadjusted Cox regression analysis, BIMA use was not significantly associated with all-cause mortality (HR: 0.95; 95% CI: 0.80 to 1.13) compared with SIMA use. The Kaplan-Meier estimates of survival are shown in Figure 1. The overall survival at 13 years was 63% (95% CI: 57 to 70) in the BIMA group and 59% (95% CI: 58 to 60) in the SIMA group (p = 0.56), and these results are shown in Table 3.

**Table 3:** Survival at 1, 5, 10, and 13 years in the overall and matched cohorts.

Overall cohort						
	SIMA			BIMA		
Time (years)	No. at risk	Survival	95% CI	No. at risk	Survival	95% CI
1	47630	0.97	0.97–0.97	538	0.96	0.94–0.97
5	35290	0.89	0.88–0.89	425	0.89	0.86–0.91
10	13380	0.72	0.71–0.72	173	0.71	0.66–0.75
13	2865	0.59	0.58–0.60	32	0.63	0.57–0.70
Matched cohort						
1	544	0.97	0.96–0.98	538	0.96	0.94–0.97
5	422	0.88	0.85–0.91	425	0.89	0.86–0.91
10	172	0.76	0.72–0.80	173	0.71	0.66–0.75
13	44	0.66	0.59–0.72	32	0.63	0.57–0.70

#### Adjusted analysis

In the age- and gender-adjusted Cox regression analysis, BIMA use was not significantly associated with mortality (HR: 1.12, 95% CI: 0.94 to 1.32) compared with SIMA use. In the final multivariable model, BIMA use was not associated with mortality (HR: 1.16, 95% CI: 0.97 to 1.37) compared with SIMA use. We introduced the propensity score for BIMA use into the full multivariable model. The propensity score was first included as a continuous covariate, and thereafter, quintiles of the propensity score were used for stratification. The HRs and 95% CIs for the association between BIMA use and mortality for both models including the propensity scores were essentially unchanged (HR: 1.20, 95% CI: 0.86 to 1.69 and HR: 1.20, 95% CI: 0.85 to 1.69) compared with the models without propensity scores.

#### Missing data and complete case analysis

In addition to the main analysis of the multiple imputed data, we performed a complete case analysis using only observations with complete information for all covariates included in the multivariable regression model (n = 19729). The result was similar (HR: 1.23, 95% CI: 0.90 to 1.67) compared with the results from the imputed dataset for the association between BIMA use and mortality.

### Association between BIMA use and the composite endpoint of all-cause mortality or rehospitalization

**Figure 2:**
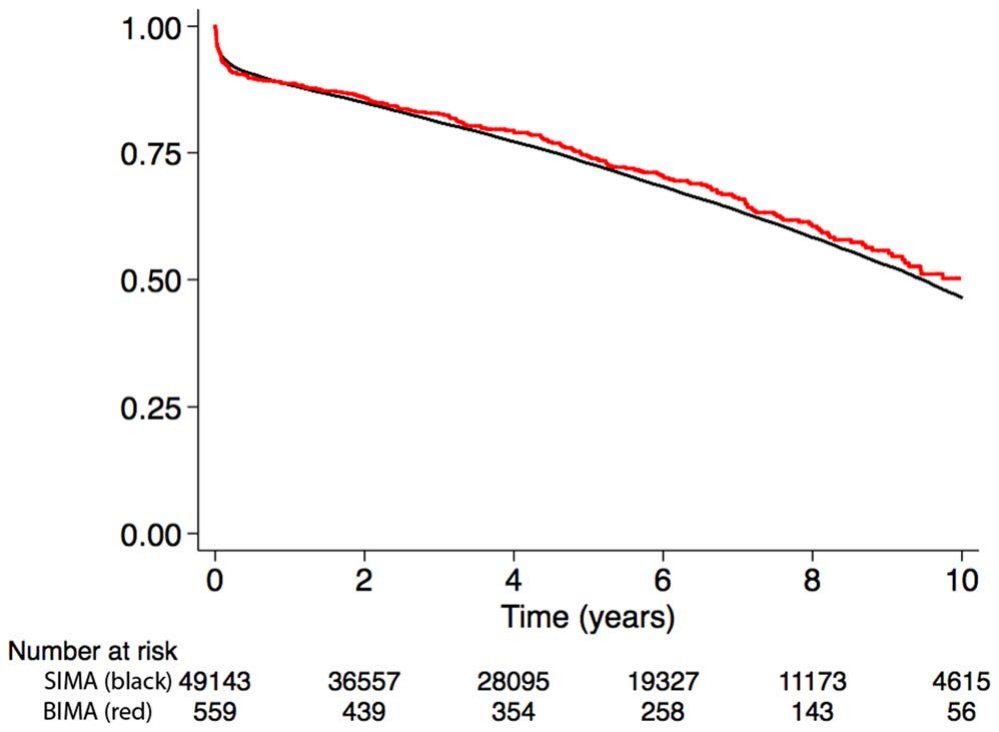
Cumulative incidence for the composite end-point all-cause mortality or rehospitalization for myocardial infarction, heart failure, or stroke in the overall cohort.

The composite all-cause mortality or rehospitalization for myocardial infarction, heart failure, or stroke occurred in 200 (36%) patients in the BIMA group, compared with 18071 (37%) in the SIMA group. The cumulative incidence of the composite endpoint at 10 years was comparable between the BIMA and SIMA group (50% vs. 47%, p = 0.20) in the overall cohort (Figure 2 and Table 4). The crude and multivariable adjusted associations between BIMA use and the composite endpoint of all-cause mortality or rehospitalization are shown in Table 5. In the final multivariable model, BIMA use was not associated with an increased risk of death or rehospitalization (HR: 1.05, 95% CI: 0.91–1.21) compared with SIMA use.

**Table 4:** Cumulative incidence of the composite end-point at 1, 5, and 10 years in the overall and matched cohorts.

Overall cohort						
	SIMA			BIMA		
Time (years)	No. at risk	Cum. incidence	95% CI	No. at risk	Cum. incidence	95% CI
1	40889	0.88	0.88–0.89	483	0.89	0.86–0.91
5	23569	0.73	0.72–0.73	313	0.74	0.70–0.78
10	4616	0.47	0.46–0.47	57	0.50	0.44–0.56
Matched cohort						
1	477	0.88	0.85–0.91	483	0.89	0.86–0.91
5	311	0.74	0.70–0.77	313	0.74	0.70–0.78
10	78	0.51	0.44–0.56	57	0.50	0.44–0.56

**Table 5:** Crude and multivariable adjusted association between BIMA use and a composite endpoint of death or rehospitalization for myocardial infarction, heart failure, or stroke in 49702 patients who underwent primary isolated non-emergent CABG during 1997 to 2008 in Sweden.

	SIMA*	BIMA
Number of patients	49143	559
Number of events (%)	18071 (37)	200 (36)
	HR (95% CI)	
Crude	1.00	0.91 (0.79–1.05)
Adjustment for age and sex	1.00	1.02 (0.89–1.18)
Multivariable model**	1.00	1.05 (0.91–1.21)

Reference category.

Multivariable adjustment was made for age, gender, estimated glomerular filtration rate, left ventricular ejection fraction, diabetes mellitus, chronic obstructive pulmonary disease, peripheral vascular disease, preoperative myocardial infarction, stroke, heart failure, perioperative acute kidney injury, and the use of cardiopulmonary bypass during surgery.

BIMA = bilateral internal thoracic artery, CABG = coronary artery bypass grafting, CI = confidence interval, HR = hazards ratio, SIMA = single internal thoracic artery.

### Secular trends in BIMA use in Sweden between 1997 and 2008

The secular trends in BIMA use in Sweden between 1997 and 2008 are presented in Table 6. We found no indication that BIMA use was increasing during the study period. The use of BIMA was also similar between the eight different cardiac surgery centers in Sweden (Table 7).

**Table 6:** Yearly use of BIMA in non-emergent primary isolated CABG during 1997 to 2008 in Sweden.

	SIMA	BIMA
Year	Number of patients (%)	Number of patients (%)
1997	4032 (98.90)	45 (1.10)
1998	4592 (98.58)	66 (1.42)
1999	4516 (98.84)	53 (1.16)
2000	4640 (98.79)	57 (1.21)
2001	4699 (98.51)	71 (1.49)
2002	4655 (98.87)	53 (1.13)
2003	4318 (98.65)	59 (1.35)
2004	4156 (99.24)	32 (0.76)
2005	3602 (99.04)	35 (0.96)
2006	3463 (99.06)	33 (0.94)
2007	3351 (98.85)	39 (1.15)
2008	3119 (99.49)	16 (0.51)
Total	49143 (98.88)	559 (1.12)

BIMA = bilateral internal mammary artery, CABG = coronary artery bypass grafting, SIMA = single internal mammary artery.

**Table 7:** BIMA use in non-emergent primary isolated CABG per cardiac surgery center during 1997 to 2008 in Sweden.

	SIMA	BIMA
Center	Number of patients (%)	Number of patients (%)
1	6588 (98.80)	80 (1.20)
2	5874 (99.12)	52 (0.88)
3	5174 (98.38)	85 (1.62)
4	3085 (99.04)	30 (0.96)
5	6955 (99.16)	59 (0.84)
6	10073 (99.16)	133 (1.30)
7	4453 (98.96)	47 (1.04)
8	6941 (98.96)	73 (1.04)
Total	49143 (98.88)	559 (1.12)

BIMA = bilateral internal mammary artery, CABG = coronary artery bypass grafting, SIMA = single internal mammary artery.

### Association between BIMA use and outcomes in the propensity score-matched cohort

By using propensity score matching methods, a satisfactory balance regarding baseline characteristics was achieved between the two treatment groups (Table 8). There was no significant association between BIMA use and early mortality (OR: 3.33, 95% CI: 0.92 to 12.1), reoperation for sternal wound complications (OR: 0.82, 95% CI: 0.49 to 1.36), or reoperation for bleeding (OR: 0.70, 95% CI: 0.45 to 1.10).

**Table 8:** Baseline characteristics in the propensity score-matched groups (n = 558 in each group) and the standardized differences between the treatment groups.

	SIMA	BIMA	Standardized difference (%)
Age, mean, years	64.5	64.4	1.5
Female sex (%)	28.1	25.8	5.3
Estimated glomerular filtration rate:			
>60 mL/min/1.73 m^2^	80.2	79.5	1.7
45–60 mL/min/1.73 m^2^	15.2	16.0	−2.2
30–45 mL/min/1.73 m^2^	4.3	3.9	1.6
>30 mL/min/1.73 m^2^	0.4	0.6	−2.8
Diabetes mellitus (%)	18.4	13.7	13.6
Hypertension (%)	53.3	51.3	4.1
Hyperlipidemia (%)	58.5	58.0	0
Peripheral vascular disease (%)	12.9	13.6	−1.9
COPD (%)	3.0	4.3	−6.2
Prior hospitalization for:			
Myocardial infarction (%)	40.5	42.8	−4.7
Stroke (%)	2.5	3.9	−7.4
Heart failure (%)	2.2	2.7	−3.3
Left ventricular function:			
Ejection fraction >50% (%)	71.1	70.3	1.7
Ejection fraction 30–50% (%)	24.3	25.4	−2.5
Ejection fraction <30% (%)	4.6	4.3	1.6
Surgery within 7 days of decision (%)	31.0	32.3	−2.7
CABG with cardiopulmonary bypass (%)	77.6	81.5	−10.2
No. of grafted coronary arteries (mean)	3.2	3.1	12.1
Radial artery use (%)	11.8	16.3	−12.1
Acute kidney injury (%)*	12.5	9.2	11.3

CABG = Coronary artery bypass grafting, COPD = chronic obstructive pulmonary disease, SD = standard deviation.

Acute kidney injury was defined as a >0.3 mg/dL (26 µmol/L) increase in postoperative creatinine values.

#### Association between BIMA use and all-cause mortality in the matched cohort

**Figure 3:**
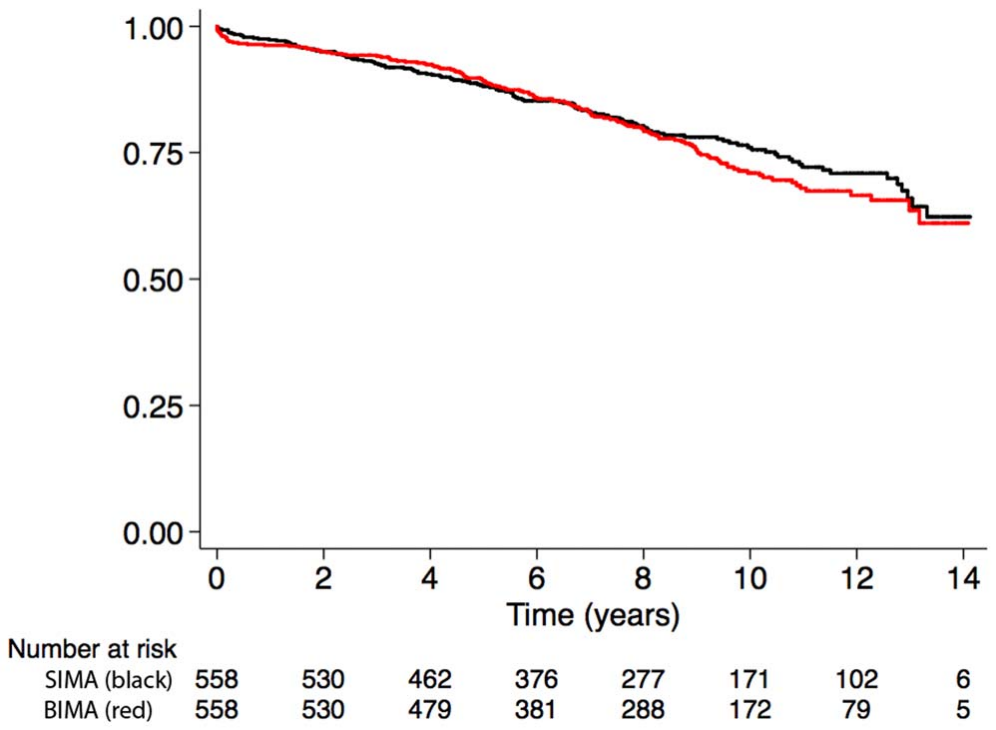
Kaplan-Meier survival in the matched cohort.

Survival at 13 years was similar between the BIMA and SIMA groups (64% vs. 66%, p = 0.47) in the propensity score matched cohort (Figure 3 and Table 3). There was no significant association between BIMA use and long-term survival (HR: 1.04, 95% CI: 0.78 to 1.40).

#### Association between BIMA use and the composite endpoint of all-cause mortality or rehospitalization in the matched cohort

**Figure 4:**
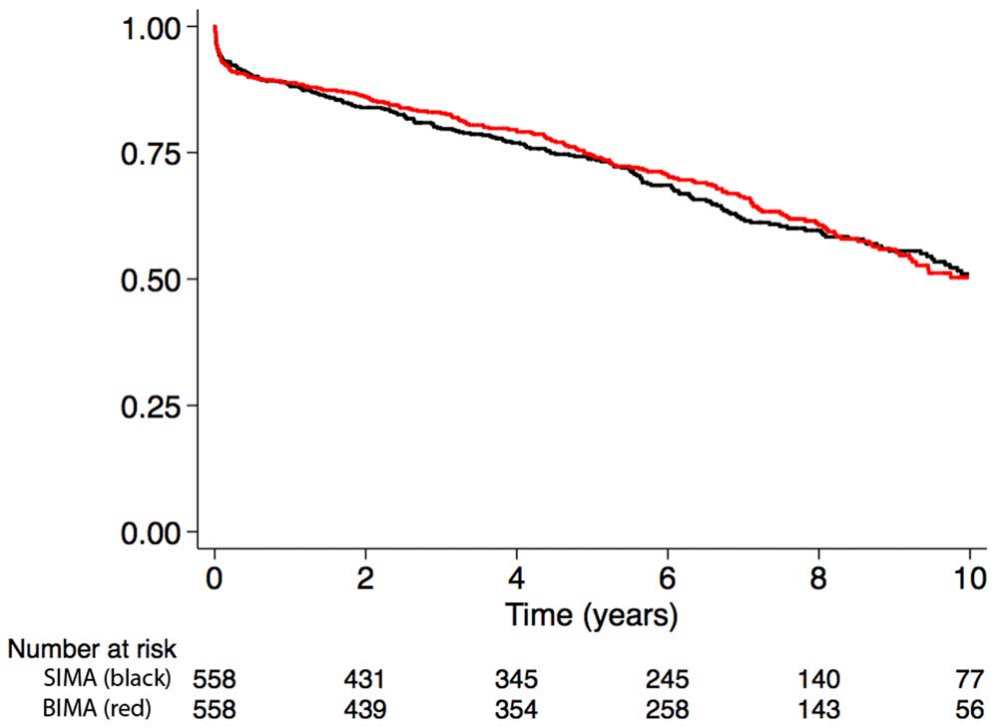
Cumulative incidence for the composite end-point all-cause mortality or rehospitalization for myocardial infarction, heart failure, or stroke in the matched cohort.

The cumulative incidence of the composite of all-cause mortality or rehospitalization for myocardial infarction, heart failure, or stroke at 10 years was comparable between the BIMA and SIMA group (50% vs. 51%, p = 0.57) in the propensity score matched cohort (Figure 4 and Table 4).

There was no significant association between BIMA use and the composite all-cause mortality or rehospitalization (HR: 0.85, 95% CI: 0.67 to 1.08).

## Discussion

We found that BIMA grafting was performed infrequently and was not associated with better long-term survival or the composite endpoint of rehospitalization for myocardial infarction, heart failure, stroke, or death from any cause compared with SIMA grafting in all patients undergoing non-emergent primary isolated CABG in Sweden during 1997–2008. The results were similar after propensity score matching, showing that BIMA grafting was not associated with better outcomes compared with SIMA grafting. The strengths of our study include the large study population and the complete and accurate follow-up and survival ascertainment due to the high-quality national Swedish registers.

However, these results are in conflict with prior studies suggesting improved outcomes in patients receiving BIMA compared with patients receiving SIMA grafting [1]–[6], [12]. Lytle et al. reported improved survival after BIMA grafting in 10124 (8123 SIMA and 2001 BIMA) patients undergoing primary isolated CABG between 1971 and 1989 who were followed for a mean of 16.5 years [2]. Improved survival in the BIMA group was sustained after propensity matching of 1152 pairs, with an increased benefit of BIMA grafting through 20 postoperative years. Survival in the BIMA and SIMA groups at 15 and 20 years was 67% versus 58% and 50% versus 37%, respectively.

In a systematic review of seven observational studies including 15962 patients (11269 SIMA and 4693 BIMA) matched or adjusted for baseline characteristics, survival was better in patients receiving BIMA, with a hazard ratio for death of 0.81 [1]. Following this systematic review, a number of observational studies supporting the use of BIMA grafting have been published [3]–[6], [12], [13].

Prior studies have questioned the use of BIMA grafting in patients with diabetes mellitus and reported a higher incidence of deep wound infection in this subgroup of patients [23], [24]. However, this finding has been reevaluated in recent studies supporting the use of BIMA in diabetic patients [5], [7], [25]. Recently, Dorman et al reported 1107 patients with diabetes mellitus undergoing CABG with BIMA or SIMA grafting [7]. In an analysis of 414 propensity-matched pairs, long-term survival was significantly improved in the BIMA group compared with the SIMA group (median 13.1 vs. 9.8 years), without any significant increase in perioperative mortality or morbidity, including sternal wound infection.

The only randomized trial comparing survival after BIMA versus SIMA grafting, the Arterial Revascularisation Trial [8], has recently completed the inclusion of 3102 patients (1554 SIMA and 1548 BIMA) who underwent isolated primary CABG. Initial results have shown no difference between the two treatment groups in 30-day or one-year mortality, or in the rate of stroke, myocardial infarction, or repeat revascularization [8]. However, an increased need for sternal wound reconstruction was observed in the BIMA group, compared with the SIMA group (1.9% vs. 0.6%). There are several possible reasons why our results were in contrast with multiple prior studies. The survival benefit of BIMA increases with time, and follow-up times were longer in other reports [5], [6], [12], [23], [26]. For example, Carrel et al reported that the use of BIMA did not improve survival in patients followed up to eight years after CABG [27]. Of note, BIMA grafting was performed in only 1% of the patients undergoing CABG in Sweden during the study period, and this situation may have influenced the results. BIMA grafting is a more technically challenging procedure than SIMA grafting and should be performed frequently by the surgeon to overcome the learning-curve and achieve the best possible results.

A second possible reason why the results were in conflict with prior studies may be due to selection bias, which is the main concern in observational studies in which patients are not randomized to treatment. The BIMA and SIMA groups were not balanced regarding the potentially confounding factors of diabetes mellitus, peripheral vascular disease, and the use of off-pump coronary artery bypass surgery. Diabetes mellitus was more common in the SIMA group, while peripheral vascular disease and the use of off-pump coronary artery bypass surgery was more common in the BIMA group. The fact that diabetes mellitus was more common in the SIMA group could be explained by a reluctance of surgeons to perform BIMA grafting in patients with diabetes due to reports of a higher incidence of deep sternal wound infections [23], [24].

The reasons why the incidence of peripheral vascular disease and the use of off-pump CABG were more common in the BIMA group are not clear. However, patients with peripheral vascular disease have a higher frequency of aortic atherosclerosis, which could have influenced the surgeon's choice of procedure. In CABG with the use of aortic cross-clamping, atherosclerotic plaques in the ascending aorta increase the perioperative stroke risk. Therefore, in patients with ascending aortic atherosclerosis off-pump CABG is preferred with the “no touch” technique regarding the aorta and BIMA in situ grafting. The higher number of patients with peripheral vascular disease and off-pump CABG may therefore indicate more pronounced general atherosclerotic disease in patients undergoing BIMA grafting.

Another possible explanation for the lack of survival benefit in the BIMA group in our study could be that the second IMA was not used in an ideal fashion. The best use of a second IMA is controversial, as discussed in a systematic review comparing BIMA and SIMA CABG [1]. These concerns regarding lower IMA patency if placed to the right coronary artery also dictated the grafting strategy in the randomized trial in which both IMA were used as grafts to the most important left-sided coronary arteries [8]. However, recent studies have shown similar survival in patients after BIMA CABG, independent of whether the second IMA was used as a conduit to the right coronary artery or the circumflex system [28], [29]. It should be noted as a limitation to our study that we were unable to analyze the influence of bypass graft configuration and target vessels on study outcomes because this information was not available in the national registers.

After adjustment for baseline characteristics, including diabetes mellitus, peripheral vascular disease, and off-pump coronary artery bypass surgery, BIMA use was not associated with mortality or the composite endpoint of all-cause mortality or rehospitalization due to cardiovascular causes compared with SIMA use.

This result is contradictory to the findings by Berreklouw and coworkers, who concluded that the positive influence of BIMA is more important for event-free survival than survival per se [30].

To further reduce selection bias, we used propensity score methods for regression adjustment, stratification, and finally, matching. The results after including the propensity scores for BIMA use into the full multivariable model were similar compared with the multivariable model without the propensity scores, with no statistically significant association between BIMA use and mortality, the composite endpoint of all-cause mortality, or rehospitalization due to cardiovascular causes compared with SIMA use. We also constructed a propensity score-matched cohort by pairing patients receiving BIMA and patients receiving SIMA. By using propensity score matching methods, a satisfactory balance regarding baseline characteristics was achieved between the two treatment groups. These results reduce the probability that the findings of the study are due to selection bias. However, due to the substantial difference in the number of patients in the two different groups, the possibility that the findings are due to selection bias cannot be excluded. The two groups were heterogeneous in some aspects, including the important and potentially confounding factors diabetes mellitus, peripheral vascular disease, and use of off-pump coronary artery bypass surgery. The differences regarding these factors could possibly have influenced the results of the study.

Another limitation that should be addressed is the high number of missing data for the preoperative variables of left ventricular function, diabetes mellitus, peripheral vascular disease, eGFR, and acute perioperative kidney injury. To retain statistical power and reduce the selection bias that may occur when deleting observations with missing covariates, multiple imputation was used to handle missing data [31]. In addition, we performed a complete case analysis using only observations with complete data. The results for the association between BIMA use and mortality from the main analysis of the multiple imputed data and the complete case analysis were very similar.

Our data clearly showed that BIMA grafting was performed infrequently and that the practice was not increasing in Sweden during the study period. Prior studies indicate superior results after BIMA grafting, and recent American and European revascularization guidelines encourage the use of BIMA CABG in suitable patients [9], [10].

### Conclusions

BIMA grafting was performed infrequently and was not associated with better outcomes compared with SIMA grafting in patients undergoing non-emergent primary isolated CABG in Sweden during 1997–2008.
